# Implications for social work teaching and learning in Universiti Sains Malaysia, Penang, due to the COVID-19 pandemic: A reflection

**DOI:** 10.1177/1473325020973308

**Published:** 2021-03

**Authors:** Azlinda Azman, Paramjit Singh Jamir Singh, Ali Isahaque

**Affiliations:** Social Work Programme, School of Social Sciences, Universiti Sains Malaysia, Penang, Malaysia

**Keywords:** COVID-19, Malaysia, social work, social work teaching, social work learning

## Abstract

The global lockdown due to COVID-19 is a major concern as all higher educational institutions face disruption in teaching, learning and assessment. Social work educators in Malaysia’s higher educational institutions are not spared of this disruption. Conventional teaching methods are now being replaced by non-conventional modes of teaching, which include online teaching and assessment using various platforms such as Zoom, WebEx and others. In embarking on online methods of teaching, social work educators will have to undergo many changes. It is particularly so as social work has a practice component that involves field training, which will be a different challenge to educators and students in this new and unexpected environment. This paper aims to discuss the implications of COVID-19 on the changes that have taken place in social work teaching and learning in Malaysia and potential responses.

## Introduction

The first three Covid-19 cases in Malaysia were reported on 25 January 2020 which involved tourists from China who arrived in Johor, Malaysia, from Singapore on 23 January 2020 (Pfordten and Ahmad, 2020). Within three (3) months, the Ministry of Health, Malaysia officially reported on 25 April 2020 that the number of Covid-19 cases in the country has increased to 5,742 with 98 fatal cases (Bedi, 2020). Studies have found that the novel coronavirus is highly contagious and has spread widely to almost all countries in the world, with the number of those infected predicted to be reaching millions worldwide. There is yet to be a cure or vaccine for the highly contagious Covid-19. While medical researchers are scrambling to develop a vaccine and drugs to contain the infection, it is expected that a vaccine would only be available for the public next year. Such concern has forced the world to be at a standstill, as most countries have imposed lockdowns to contain the spread of this highly contagious disease. Subsequently, the World Health Organisation (WHO) projected that without a publicly available vaccine, the Covid-19 virus would threaten the world’s population (Mok, 2020).

Covid-19 pandemic has an immense effect on how people live all around the world. The enforcement of lockdowns has severely affected industries, including education, as schools and universities all around the world were forced to close their campuses and this subsequently, disrupts the learning of millions of students (Rahman, 2020). Similarly, in Malaysia, the government-enforced movement control order (MCO) has forced the closure of all public and private educational institutions. The administration of Universiti Sains Malaysia (USM) announced a temporary closure of all of its campuses beginning 17 March 2020, affecting close to 30,000 students even before the nation’s MCO was implemented on 18 March 2020 (Universiti Sains Malaysia, 2020). As the students and lecturers are not able to attend face-to-face classes, more of online remote learning to accommodate teaching and learning during the MCO was mandated. This initiative has allowed students, including from the social work programme, to attend classes virtually.

## The social work programme in USM

USM is the first university to offer a Bachelor of Social Work programme in Malaysia. It is a four-year generic programme to train professional social workers. This is in line with the government’s aspiration of providing professional social welfare services to facilitate social development in Malaysia. The primary outcome of this programme is to develop social work graduates who possess the adequate practical and theoretical knowledge of social work, as well as a sound understanding of the social work philosophy and skills, which are needed for professional and accountable social work practices. At present, there are 300 full-time students enrolled year one to year four of the bachelor programme.

## Adaptations and challenges

Undoubtedly, the COVID-19 global pandemic has had a significant impact on the delivery of the USM Social Work Bachelor programme. Despite the lockdown, all the ten social work educators involved in this programme are committed to the teaching process to ensure that the pandemic does not disrupt students’ learning. The subsequent sections detail the USM social work educators’ experiences and challenges in facilitating the teaching and learning process during the lockdown enforced to contain the spread of Covid-19.

## Teaching

While the beginning of the second semester started in February 2020, the teaching and learning schedule of the Social Work Programme was disrupted by the announcement of the two-week MCO enforced by the Malaysian government, which began on 18 March 2020. Initially, the government announced that the MCO last only two weeks. It was subsequently extended three times. The current MCO will end on 12 May 2020 and with the possibility for further extension. As a result of the MCOs, USM was forced to close all of its campuses, and face-to-face lectures were suspended.

In early April 2020, USM announced that the second semester would commence and all courses, including social work courses, will be taught entirely through virtual and remote online learning in response to the uncertainty over the duration of the MCO. It was an immediate demand for all of the social work educators to make changes to their lessons plan by incorporating online teaching and assessment to ensure the smooth flow of the curriculum content. Among the standard tools used include WebEx, Loom, Zoom as well as audio recordings of lectures. In addition, in support of online learning, all of the materials for the course, such as lecture notes, have been made available to students prior to the online sessions via the student’s e-learning portal. This has allowed social work students to read and study prior to the online learning sessions and to obtain further clarification of the subject matters with their lecturer during the online sessions. In order to ensure all students received the learning materials, the educators have also gone the extra mile to courier printed hard copy learning materials as well as the compilation of the softcopy in a thumb drive for students, particularly those living in rural areas, who may have no or very limited access to the internet.

## Assessment

Traditionally, a 40:60 marks allocation was set for most of the courses in social work. This means that 40% of the students’ marks will be based on assignments, and the final exam will determine 60% of their marks. However, due to campus closure, all tests and exams for Semester II, Academic Session 2019/2020 will be replaced with formative and summative assessments. In this regard, students’ grades will be entirely determined by coursework components, including online presentations, open-book tests, quizzes, casework, case studies, assignments, short essays, and other forms of assessments deemed suitable. All of these assessment tasks will be assigned through the students’ e-learning portal. All of the assessments assigned to the students are based on programme and course learning objectives, Bloom’s taxonomy, and outcome-based education. Every course assessment was discussed and vetted among the social work educators before it was assigned to the students. This is to ensure that all class activities were in line with the targeted programme outcome.

Such an adaptation process requires the social work educators to go through the process of vetting the coursework assignments or activities and deciding the allocation of marks for each assessment. With no final exam, the coursework components need to be vetted to ensure all of the adjusted assessments are in line with the targeted taxonomy and course objectives. To adhere to the social distancing rules, the vetting process was conducted through WebEx and Zoom. Although online mechanisms were employed for the vetting process, the educators will also have to take into account every measure for confidentiality.

## Field practice

A critical challenge would be the running of social work field practice, which is mandatory for all social work programmes. The on-going lockdown has forced most social work agencies to cease operations. Hence, the programme had the most challenging situation in the postponement of students who are currently doing their field practice. The implication to the postponement is inadequate field practice hours falling short of the standard requirement for graduating. This requires the programme to reorganise the field practice structure with immediate effect in order to cater to the unforeseen crisis. The main concern involves determining activities or assessments that can be replaced according to the total hours of field practice not yet completed. This has created another level of strain on the programme and students. A decision has to be made immediately in order to consider the different types of activities that can gear the students with the primary objectives of field practice in conducting casework, group work as well as community work.

## Reflection on educators’ experiences

Social work educators faced numerous challenges in conducting online learning this semester. This is because this form of learning is a novel and alternative approach to teaching. Many of the social work educators do not have sufficient technical skills and confidence to conduct online teaching. Thus, the new skills required for the implementation of remote learning have to be learned quickly and effectively. This, of course, has created some stress among the many educators due to these unanticipated changes.

Although online teaching is not unknown in Malaysian educational institutions, it is not the norm nor widely used. Instead, the conventional method or teacher-centred pedagogy is the default and more effective approach, or rather the educational culture in most learning institutions, not only in teaching social work but in many other disciplines as well. The nationwide closure of education premises forced these institutions to embark on online teaching. Online teaching courses are made available to all educators, and many have chosen to attend them to improve their knowledge and skills.

For several educators who have low to poor internet access, the immediate option available to them is to deliver their lectures via low bandwidth ways of communicating like Instagram and WhatsApp Messenger, while quickly learning other effective methods of lecture delivery. These challenges have also placed the educators in stressful situations as they too have to juggle with their challenges of working from home.

The traditional routine teaching and learning all this while has made many social work educators complacent, and thus online learning becomes an additional burden. The crisis situation has indirectly pushed educators to embark on online learning instantly. However, support from other educators familiar with online learning methods has helped ease their worries. Unintentionally, these changes support the idea of using blended learning as a long-term aim of the programme and the university as a whole.

The current changes in teaching and learning online has raised the primary question as to the fear of no longer being able to conduct face-to-face learning which is seen to be more effective. This is particularly critical as social work focuses not only on “learning the knowledge” but also to “learning what to do”, which is connected to direct practice and involves conducting casework, group work and community work. Many of the social work educators felt that, in social work, it is critical to continue with face-to-face learning and be supported by online learning in some parts of the course content. Therefore, it is hoped that the crisis will end soon in order to allow social work graduates to acquire first-hand skills or the needed competencies in working with various clientele groups directly.

## Reflection on students’ experiences

Similarly, social work students face several challenges when it comes to remote learning. The most crucial issue is access to high-speed internet for such learning to take place. Based on a quick survey of all social work students from various socio-economic backgrounds, almost all (95 - 97%) of the year one to year four students have access to the internet, while only 3% to 5% have access difficulties. Although the percentages of those who have internet access problems are considered low, still immediate measures have been taken in order to ensure all students have equal access to learning. This requires social work educators to offer all types of teaching and learning alternatives via online ranging from high to low bandwidth methods of online sessions for easy access of learning materials including pre-recorded lectures to ensure that no students are left in the learning process.

There is still a significant disparity between accesses to the internet, especially in the rural areas of Malaysia. This has posed significant challenges for students who are required to go for online classes and examinations. Recently, a university student from a rural district in Sabah, East Malaysia, recorded her experience on social media. Spending a night on a tree to get a steady internet connection so she could attend her online examination, her video went viral. Her plight represents the often-invisible struggle of the thousands of students living in rural areas.

Students’ lack of access to devices is another significant challenge to support e-learning. The Ministry of Education (MOE) in its recent survey of approximately 900,000 students reported that 37% of students do not have access to any technological devices for learning. In this light, only 6% to 9% of students own a personal computer and/or a tablet (Department of Statistics Malaysia, 2020). The other students need to share personal computers or devices with their siblings or other family members according to the same report. Device ownership is made more challenging for students from low-middle income families as many faces financial hardship during the MCO as the unemployment rate increases. This could lead to a wider gap in access to learning as cash strapped families will prioritise putting food on the table than spending money to buy gadgets or devices. If online teaching and learning are going to be a new norm for the foreseeable future, there is a need for the government to have the policy to increase device affordability and ownership.

The practice of remote online learning has also impacted the students tremendously. Since the offering of academic courses is in the form of full mode coursework assessments, there are drastic changes in the course expectations. Initially, students were excited to be experiencing different forms of online learning. However, after a while, students begin to feel the burden by the number of assignments and assessments. All these contribute to some difficulty and put more stresses to some students. In order to avoid burnout among students, the social work educators were encouraged to negotiate among themselves in determining assignment weightage of the different social work courses as a means of minimising and spreading students’ workload. On the whole, not only educators will have to go through the drastic adjustments of teaching, but also the students to cope with the higher weightage of workload during the current pandemic.

## Conclusion

The Covid-19 pandemic has undoubtedly put our lives on hold. It has forced lockdowns and the closure of higher learning institutions. Like in many other parts of the world, online teaching became the best alternative given the circumstances. Online teaching is no longer a choice for the educators, thus requiring them to go on a steep learning curve on how they should conduct their lessons to facilitate learning despite being confined at homes. A new mantra is “work from home”. Quickly training and workshops were conducted by the respective institutions for educators on the basics of online teaching and learning. This is to ensure continuous delivery of effective learning during the lockdown period and beyond. USM’s social work programme has moved fast to prepare its academic staff and students to embrace the challenges.

Online learning is the obvious choice, and during this episode of Covid-19, educators and students are eager to embark on a new journey but at the same time are also anxious about many issues, particularly in ensuring the provision of quality social work education. Burgess and Sievertsen (2020) stated that the lockdown of educational institutions not only interposes the teaching and learning for educators and students around the globe but also revolved around accurate assessments to replace formal examinations which have been suspended temporarily. All of these are pertinent in order to ensure the social work graduates will acquire the needed competencies as required by the social work profession.

Online learning has become a ‘new’ norm throughout the lockdown, and the possibility for the many more months to come. Hence, it is hoped that all social work educators will persevere in these trying times and use all opportunities and resources to facilitate the remote teaching and learning process efficiently. Despite the MCO, teaching and learning continue, and social work educators need to work together to achieve the outcomes of producing competent social workers. While there is still uncertainty over when the threat of the Covid-19 pandemic will be over, proactive measures must always be in place to ensure that USM continues to produce a well-trained and competent social worker. Resilience and optimism are essential, and it is important to remember that this experience will pass and a new dawn is imminent, as stated by the Malaysian Prime Minister: “After the rain comes the sun.” A new tomorrow is foreseeable.
